# Posterior Gastroenterostomy: An Unusual Post-Operative Complication with Report of a Case

**Published:** 1906-04

**Authors:** Joseph Burke

**Affiliations:** Buffalo, N. Y.; Surgeon to the Emergency Hospital


### Posterior Gastroenterostomy: An Unusual Post-operative Complication with Report of a Case.[1]

BY JOSEPH BURKE, D. Sc., M. D., Buffalo, N. Y.
Surgeon to the Emergency Hospital.

OF the many operative procedures that recent surgery has given to the so-called incurable, there is none that merits consideration more than the one that cures the chronic dyspeptic—namely, gastroenterostomy, paricularly the transmesocolic operation for the relief of chronic gastric ulcer. In this as in many other operations there have been numerous failures reported in the earlier cases, the chief "bete noir" being the occurrence of regurgitant vomiting due to sagging of the jejunum where the long jejunal loop had been employed. The simultaneous enteroenterostomy by suture, or by the Murphy button, and other ingenious methods sought to correct this fault in technic and thus avoid this so-called vicious circle, but in spite of the improvement cases now and then did poorly.

In the operation as perfected by Moynihan, the short loop is used, two to four inches from the plica duodenalis and the anastomosis is made at the lowest point of the stomach, and in these cases regurgitant vomiting has become a thing of the past. Some surgeons, for example Deaver, still cling to the long loop with enteroenterostomy, claiming good results. Inasmuch as I have used both the long loop with enteroanastomosis and the short loop methods in a number of cases, I believe that the latter being less time consuming and simpler, as well as it is mechanically ingenious, is the preferable one.

Although sagging of the long loop of the jejunum has been held responsible for many failures of gastroenterostomy in which regurgitant vomiting has occurred, yet this is not true in all cases as the following unique one that I operated at the Emergency Hospital will aptly illustrate,—a case wherein an unlooked-for post-operative complication occurred.

CASE: M. Mc., 24 yrs. Irish, single. Patient enjoyed good health until about three years ago when she began to complain of gastric disturbance; she first experienced severe pains in the epigastrium from immediately after to one hour after eating, which would persist until vomiting occurred; pain was always more intense when vomiting did not take place; patient would feel exhausted at this time. The vomited matter was very sour, described as "sour, hot, and burning." Pains soon became so great that patient was obliged to take to her bed for a month, during which period scarcely any food was retained.

On two occasions she suffered from sudden severe excruciating pain in the stomach and extreme nausea, but no vomiting took place; hot applications failed to give the necessary relief and hypodermics of morphia became a necessity. Six months ago I saw the patient in an attack of what I then diagnosticated as an acute perforation of a gastric ulcer; sudden acute pain in the epigastrium, paleness, small rapid pulse (120) and respirations, rigidity of left rectus muscle, were prominent symptoms. The patient has suffered from distressing heartburn, pyrosis and persistent gaseous eructations.

PHYSICAL EXAMINATION: lungs heart and kidneys, normal. Stomach in normal position, not appreciably enlarged. Examination of stomach contents revealed marked hyperacidity and red blood cells.

OPERATION: An incision four inches long was made below the ensiform cartilage three-fourths of an inch to the right of the median line, down to and through the peritoneum. The stomach being delivered, the transverse colon was drawn upward exposing the transverse mesocolon. The posterior stomach wall was drawn through an opening made in the latter. On the posterior surface of the stomach a large adhesion to the mesocolon was found that corresponded to an ulcer of the posterior wall which had previously perforated. A gastroenterostomy by Connell suture with button enteroenterostomy was made, and the abdominal wound closed. At the end of eight days patient was able to take soft diet, that is, rice, eggs, toast, milk and broths.

After four months of absolute relief from gastric disturbance, symptoms which indicated a return of her original trouble appeared suddenly. During the four months her appetite was ravenous, her digestion of mixed diet perfect; stool was daily and regular; the patient gained thirty pounds; no vomiting, no medication necessary. The return of the symptoms began with vomiting of a very sour liquid with food, after eating; pain in the epigastrium just to the left of the median line; she had great distress from gaseous distention. The vomiting became so persistent that even liquids would be rejected as soon as taken; the vomited matter at times was green. Evidently constriction of the gastro-anastomotic opening had taken place or probably acquired vicious circle.

The second operation revealed the following interesting conditions: the union of bowel and stomach, as well as the enteroenterostomy union was perfect. At a point half an inch away from the stomach, coming from transverse mesocolon was a tough band of adhesion one-eighth inch wide attached to and constricting the efferent jejunal loop; the gastrointestinal anastomsis opening would hardly admit the tip of the little finger; evidently the partial obstruction of the circulation of intestinal contents from the stomach to and through the efferent bowel put the new opening proportionately out of function, in consequence of which corresponding contraction took place. The anterior line of gastrointestinal union was incised, some encapsulated celluloid was removed, the interior of the stomach and bowel being exposed. The opening was made larger and the anastomosis completed as usual. Today, one year after the second operation, the patient reports perfect health.

888 MAIN STREET.

